# Association of uric acid levels with the risk of severe CED in LVO-AIS patients after mechanical thrombectomy

**DOI:** 10.3389/fneur.2026.1773323

**Published:** 2026-05-04

**Authors:** Mayila Abuduaini, Xinli Xiong, Gang Li, Tianrui Zhu, Yaling Zheng, Qi Wang, Zhengyu Huang, Yue Zhang

**Affiliations:** Department of Neurology, Shanghai East Hospital, School of Medicine, Tongji University, Shanghai, China

**Keywords:** acute ischemic stroke, cerebral edema, large vascular occlusion, mechanical thrombectomy, uric acid

## Abstract

**Objectives:**

Cerebral edema (CED) significantly exacerbates mortality in patients with acute ischemic stroke (AIS) and can offset the benefits of endovascular therapy. Uric acid (UA) is recognized for its potential neuroprotective properties. This study aimed to investigate the association between serum UA levels and moderate-to-severe CED in large-vessel occlusion (LVO-AIS) patients following mechanical thrombectomy (MT).

**Methods:**

We retrospectively analyzed 272 patients with anterior circulation LVO-AIS who achieved successful reperfusion (mTICI grade 2b-3) after MT. Patients were categorized into mild and moderate-to-severe CED groups, Multivariate logistic regression and interaction analyses were employed to determine the relationship between UA levels and the risk of moderate-to-severe CED.

**Results:**

Patients in the no-to-mild CED group exhibited significantly higher UA levels compared to the moderate-to-severe CED group (median 310.0 vs. 302.0 μmol/L; *p* < 0.05). After adjusting for confounders (hypertension history, NIHSS, TICI, and ASPECT scores), higher UA levels were inversely correlated with moderate-to-severe CED risk (adjusted OR: 0.74; 95% CI: 0.56–0.99; *p* = 0.044). Interaction analysis revealed that this protective effect was more pronounced in patients with lower admission blood glucose (<7.5 mmol/L), higher mTICI grades (grade 3), and a history of hypertension (all P-interaction<0.06). Specifically, among patients with glucose<7.5 mmol/L, those with UA ≥ 360 μmol/L had a 76% lower risk of moderate-to-severe CED (aOR: 0.24; 95% CI: 0.09–0.69; *p* = 0.033).

**Conclusion:**

Higher serum UA levels may serve as a protective factor against moderate-to-severe CED following MT in LVO-AIS patients. This association is particularly significant in patients with lower glucose levels, optimal reperfusion (mTICI 3), and a history of hypertension.

## 1 Introduction

Large vessel occlusion acute ischemic stroke (LVO-AIS) is associated with high mortality and disability rates, with the prognosis heavily reliant on timely and effective restoration of cerebral tissue perfusion. The efficacy of endovascular thrombectomy (EVT) for AIS has been established in randomized clinical trials (RCTs) (1). Despite its benefits in reducing mortality and improving functional outcomes, > 50% of patients still face a poor prognosis (2). Cerebral edema (CED) emerges as a significant complication during the early postoperative period in LVO-AIS patients. Those with CED often exhibit ischemic infarction and intracerebral hemorrhage, with mortality rates soaring to nearly 80% in severe cases (3). Following reperfusion, the extravascular movement of plasma across the compromised blood–brain barrier (BBB) induces tissue swelling, potentially leading to infarction or hemorrhage if the BBB is severely impaired, thereby exacerbating CED (4). Consequently, current research primarily focuses on reperfusion injury and brain protection post-AIS reperfusion therapy.

Multiple studies have highlighted the potential brain-protective properties of metabolic factors such as uric acid (UA) (5). UA, a byproduct of purine metabolism, stands as one of the foremost endogenous antioxidants, exerting neuroprotective effects by scavenging free radicals, curbing the inflammatory cascade, and diminishing BBB permeability (6). Furthermore, during the acute phase of ischemic stroke, serum UA levels decline, and the extent of this decline correlates positively with severe stroke, unfavorable stroke progression, large infarct volume, and poor prognosis (7). Nonetheless, the protective role of UA in AIS remains contentious, likely owing to variations in outcome evaluations, potential confounding factors, and diverse study populations. Given that CED significantly impacts patient survival and prognosis, this study centers on CED occurrence in individuals with AIS featuring large vessel occlusion who underwent mechanical thrombectomy. Additionally, we explored the influence of UA on CED and its related factors.

## 2 Methods

### 2.1 Study population

**Figure 1:**
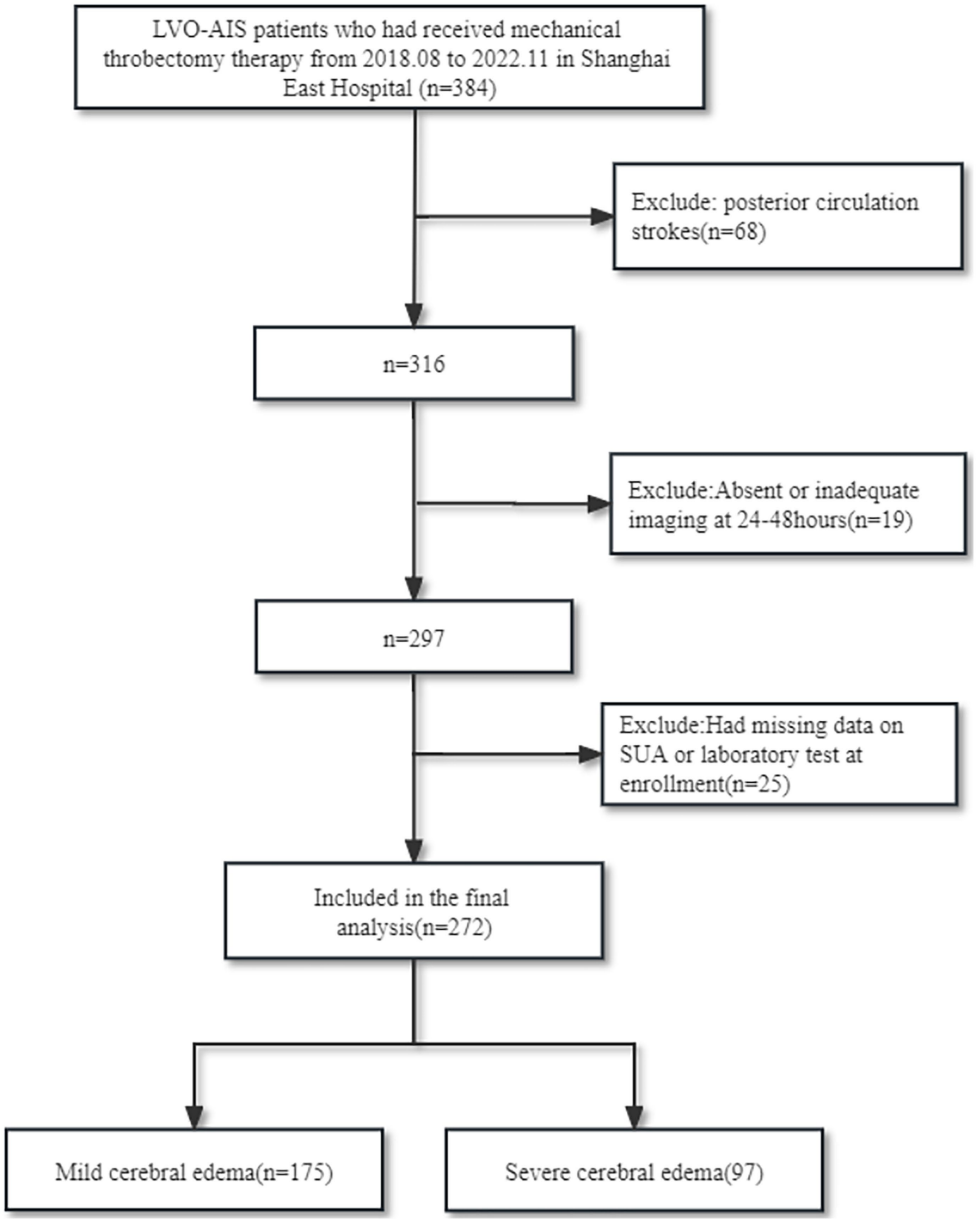
Flow chart of the study’s recruitment.

Patients with LVO-AIS at the Department of Neurology, Dongfang Hospital of Tongji University, between August 2018 and November 2022 who underwent MT and achieved an modified Thrombolysis in Cerebral Infarction (mTICI) grade of 2b-3 were included in this study. The selection criteria were as follows: (1) Patients meeting diagnostic criteria for acute cerebral infarction induced by LVO and indications for MT, excluding those < 18 years old. (2) Exclusion of patients with posterior circulation strokes. (3) Availability of complete imaging data within 24–48 h post-thrombectomy. (4) Availability of complete UA or laboratory test data at enrollment. (5) Comprehensive clinical information (Figure 1).

### 2.2 CED grade

The SITS-MOST edema scale was utilized to assess CED grade (3). Mild CED was characterized by focal brain swelling encompassing up to one-third of the hemisphere (grade 1), while moderate CED entailed focal brain swelling exceeding one-third of the hemisphere (grade 2). Severe CED was identified by focal brain swelling accompanied by midline shift (grade 3). Signs of focal CED typically included narrowing of the cerebrospinal fluid space, such as cortical sulci effacement or ventricular compression (8). To facilitate comparison, the two higher grades of the scale were amalgamated into a composite outcome. Patients exhibiting moderate-to-severe CED (SITS-MOST grade 2–3) were allocated to the moderate-to-severe CED group, whereas patients displaying no-to-mild CED (grade 0–1) were assigned to the no-to-mild CED group, based on previous research findings. CT imaging was performed within 24–48 h post-thrombectomy. Edema grading was independently performed by two neurologists experienced in neuroimaging; discrepancies were resolved through consensus discussion with a third senior neurologist. Given the retrospective design, complete blinding of assessors to serum UA levels and clinical data was not feasible, and formal inter-rater reliability statistics were not calculated.

### 2.3 Data collection and clinical variables

At admission, comprehensive clinical data were gathered, encompassing basic information such as age, sex, weight, height, and smoking history. Past medical history was recorded, including a history of coronary heart disease, hypertension, diabetes mellitus, dyslipidemia, atrial fibrillation, and previous stroke. Additionally, stroke characteristics were documented, including occlusion site, stroke-to-embolization time, Glasgow Coma Scale (GCS), mTICI grade, National Institutes of Health Stroke Scale (NIHSS), and Alberta Stroke Program Early CT Score (ASPECT). Laboratory tests encompassed white blood cell count, platelet count, UA levels, and randomized blood glucose levels, among others. Moreover, the results of electronic computed tomography (CT) scans (using detectors ranging from 64 to 320) post-mechanical thrombolytic therapy were collected.

All data were retrieved from the electronic medical record systems of participating hospitals. Serum UA levels were measured as part of the routine laboratory workup obtained upon hospital admission, prior to mechanical thrombectomy and before any major fluid resuscitation or pharmacological intervention. When multiple UA measurements were available for a given patient, the first recorded admission value was used for analysis. Fasting status at the time of blood sampling was not systematically documented in the electronic medical records, which represents a limitation of this retrospective study. All serum UA measurements were performed using an automated biochemical analyzer.

### 2.4 Statistical analysis

Continuous data are presented as medians and interquartile ranges (IQRs). The Kolmogorov–Smirnov test was used to assess normality. Group differences in continuous variables were compared using the Wilcoxon rank sum test, and categorical variables were compared using the χ^2^ test. Multifactorial logistic regression was used to identify factors associated with CED after MT. Variables with *p* < 0.05 in univariate analysis, along with clinically relevant confounders (hypertension history, NIHSS score, mTICI grade, and ASPECTS score), were included in the multivariable model. A restricted cubic spline function within a Generalized Additive Model (GAM) framework was applied to visualize the dose–response relationship between UA levels and CED risk, with results presented on the log-OR scale. Interaction analyses were performed to identify variables modifying the association between UA levels and CED, incorporating clinically significant predictors of AIS prognosis (9). All interaction analyses were considered exploratory, and no formal multiplicity correction was applied. All analyses were performed using R 4.3.2, with a two-sided significance level of 0.05.

## 3 Results

### 3.1 Comparison of baseline data between mild and moderate-to-severe CED groups

A total of 272 patients meeting the inclusion criteria participated in this study, comprising 175 patients with mild CED and 97 patients with moderate-to-severe CED. Their baseline characteristics, medical history, stroke features, and laboratory findings are presented in Table 1.

**Table 1:** Baseline characteristics of patient severents with LOV-AIS undergoing MT.

Variable	CED level		*p* value
	Mild CED group	Severe CED group	
N	175	97	
Age (median [IQR])	71.0 [64.0, 79.0]	70.0 [65.0, 76.0]	0.831
Female %	73 (41.7)	40 (41.2)	0.939
Occlusion (%)			0.399
M1	85 (48.6)	49 (50.5)	
M2	23 (13.1)	8 (8.2)	0.259
ICA	59 (33.7)	38 (39.2)	0.686
Other	8 (4.6)	2 (2.1)	0.303
Time from accident to operation/h (median [IQR])	7.0 [5.0, 13.0]	7.0 [5.0, 11.0]	0.084
TICI score (= Level 2b) %	41 (23.4)	35 (36.1)	0.027
NIHSS (median [IQR])	14.0 [10.0, 18.0]	16.0 [13.0, 18.0]	0.058
GCS (median [IQR])	14.0 [12.0, 15.0]	14.0 [12.0, 15.0]	0.632
Aspect (median [IQR])	10.0 [7.0, 10.0]	7.0 [5.0, 9.0]	<0.001
Hypertension (=Yes) %	104 (59.4)	71 (73.2)	0.024
Diabetes (=Yes) %	51 (29.1)	20 (20.6)	0.127
Dyslipidemia (=Yes) %	3 (1.7)	2 (2.1)	0.848
Atrial fibrillation (=Yes) %	56 (32.0)	34 (35.1)	0.609
Previous stroke/TIA (=Yes) %	50 (28.6)	18 (18.6)	0.070
Tobacco (=Yes) %	53 (30.3)	32 (33.0)	0.645
WBC/*10`9/L (median [IQR])	8.6 [6.9, 10.7]	10.3 [7.9, 12.3]	0.306
PLT/*10`9/L (median [IQR])	190.0 [152.5, 240.5]	179.0 [145.0, 234.0]	0.515
CREA/umol/l (median [IQR])	68.0 [54.0, 85.5]	67.0 [55.0, 86.0]	0.667
UREA/mmol/l (median [IQR])	5.2 [4.0, 6.5]	4.8 [4.0, 5.7]	0.324
UA/ummol/l (median [IQR])	310.0 [252.5, 373.5]	302.0 [230.0, 354.0]	0.046
UA > =360	53 (30.3)	19 (19.6)	0.057
Glucose level/mmol/l (median [IQR])	7.5 [6.4, 9.8]	7.6 [6.2, 9.3]	0.540

No statistically significant differences (*p* > 0.05) were observed between the two groups regarding occlusion site, time from symptom onset to thrombus retrieval, GCS score, history of diabetes mellitus, dyslipidemia, atrial fibrillation, stroke history, and smoking history. Similarly, no statistical differences were observed (*p* > 0.05) in leukocyte counts, platelet counts, creatinine, urea, and randomized blood glucose levels upon admission between the two groups.

Baseline NIHSS scores were higher in the moderate-to-severe CED group compared to the no-to-mild CED group (median [IQR] = 16.0 [13.0, 18.0] vs. 14.0 [10.0, 18.0]), although this difference did not reach statistical significance (*p* = 0.058). A higher prevalence of hypertension was observed among patients in the moderate-to-severe CED group compared to the no-to-mild CED group, which was statistically significant (*p* < 0.05). Additionally, patients in the moderate-to-severe CED group exhibited lower mTICI classification and ASPECT scores compared to those in the no-to-mild CED group, with statistically significant differences observed between the two groups (*p* < 0.05).

When considering UA as a continuous variable, patients in the no-to-mild CED group exhibited significantly higher UA levels (median [IQR] = 310.0 [252.5, 373.5]) compared to those in the moderate-to-severe CED group (median [IQR] = 302.0 [230.5, 354.0]), with a statistically significant difference observed between the two groups (*p* < 0.05).

Alternatively, when categorizing UA using a clinical threshold of 360 μmol/L, it was found that a greater proportion of patients (53 out of 175, 30.3%) in the no-to-mild CED group had higher UA levels compared to the moderate-to-severe CED group. However, this difference did not reach statistical significance (*p* = 0.057). (Table 1).

### 3.2 Multifactorial logistic regression analysis of UA levels and CED

When examining UA levels as a continuous variable, a significant negative association was observed before adjusting for potential confounders, with an OR of 0.77 (95% CI, 0.59–1.00; *p* = 0.046) for a one standard deviation increase in UA levels (Supplementary Figure S1A and Table 2). After adjusting for potential confounders such as hypertension, mTICI grades, NIHSS scores, and ASPECT scores at baseline—indicators with a *p*-value < 0.05 in univariate analysis—a significant negative association between UA levels and the risk of CED persisted, with an adjusted OR of 0.74 (95% CI, 0.56–0.99, *p* = 0.044) (Supplementary Figure S1B and Table 2). When UA levels were categorized based on a clinical threshold and adjusted for potential confounders, patients with UA levels ≥ 360 μmol/L were found to have a lower risk of moderate-to-severe CED compared to those with UA levels < 360 μmol/L (adjusted OR [aOR], 0.53; 95% CI, 0.28–1.02; *p* = 0.057). However, these results were not statistically significant when UA levels were categorized according to the clinical threshold of 360 μmol/L (Table 2). The threshold of 360 μmol/L was selected based on its correspondence to the hyperuricemia criterion for females in Chinese clinical practice guidelines and its use in prior stroke-related UA studies. We acknowledge that sex-specific thresholds (>420 μmol/L for males; >360 μmol/L for females) may be more appropriate and should be explored in future larger studies.”

**Table 2:** Logistic regression analysis of factors influencing CED in AIS patients undergoing MT.

Variable	N	Severe CED, *n* (%)	Univariate analysis		Multivariate analysis	
			OR (CI)	*p* value	OR (CI)	*p* value
Continuous, Per SD	272	97(35.7)	0.77 (0.59,1.00)	0.046	0.74 (0.56,0.99)	0.044
UA						
<360	200	78 (39.0)	ref		ref	
> = 360	72	19 (26.4)	0.56 (0.31,1.02)	0.057	0.53 (0.28,1.02)	0.057

Adjusted for “Hypertension,” “TICI,” “NIHSS,” “Aspect”.

### 3.3 UA and CED correlation curves

A graph depicting the relationship between UA and CED demonstrates a generally inverse, non-linear trend, indicating a tendency toward decreasing CED risk with increasing UA levels. This trend is evident before adjusting for potential factors (such as history of hypertension, NIHSS score, mTICI score, and ASPECT score) that had *p* < 0.05 in one-way analysis. Subsequently, after adjusting for these potential factors, the relationship between UA and CED remained, with a more pronounced curvature in the curve, suggesting a stronger correlation (Supplementary Figure S1).

### 3.4 Interaction analysis between clinically important variables

#### 3.4.1 Interaction analysis when UA level is used as a continuous variable

The following interaction analyses were pre-specified based on established biological and clinical rationale and should be considered exploratory. No formal correction for multiple comparisons was applied; results should therefore be interpreted with caution. The following interaction analyses were pre-specified based on established biological and clinical rationale and should be considered exploratory. No formal correction for multiple comparisons was applied; results should therefore be interpreted with caution. To evaluate the impact of UA levels on CED, clinically significant variables influencing AIS prognosis and UA levels were incorporated. The interaction between UA levels and CED was examined to identify factors affecting this relationship.

The results of interaction analysis of significant covariates, after adjusting for confounders such as history of hypertension, NIHSS score, TICI score, and ASPECT rating—variables with *p* < 0.05 in univariate analysis—revealed that when UA levels were analyzed as a continuous variable and glucose levels were categorized based on the median (7.5 mmol/L), patients with lower baseline glucose levels (< 7.5 mmol/L) exhibited a more robust negative association between UA levels and CED (multivariable-adjusted interaction *p* = 0.058). Similarly, a stronger negative association between UA levels and CED was observed in patients with a higher baseline mTICI grade (grade 3) (multivariable-adjusted interaction *p* = 0.029). Additionally, patients with a history of hypertension displayed a stronger negative association between UA levels and CED (multivariable-adjusted interaction p = 0.02), as outlined in Table 3. Higher UA levels, potentially acting as a protective factor against CED development in patients with LVO-AIS after MT, appeared to exert a more pronounced effect in patients with lower blood glucose levels, higher mTICI grades, and a history of hypertension. Notably, no significant interaction was observed for the remaining variables, including age, sex, history of diabetes mellitus, NIHSS score, ASPECT score, creatinine, and urea (multivariate-adjusted interaction *p* > 0.05).

**Table 3:** Interaction analysis between significant variables when uric acid level is used as a continuous variable.

Subgroup	UA			P for interaction
	N	Severe CED, *n* (%)	OR (95%CI)	
Age (Median)				0.763
<70	130	45(34.6)	0.69 (0.45,1.04)	
≥70	142	52(36.6)	0.73 (0.48,1.13)	
Gender				0.720
Male	159	57(35.8)	0.70 (0.47,1.04)	
Female	113	40(35.4)	0.79 (0.51,1.23)	
Hypertension				0.020
No	97	26(26.8)	1.19 (0.71,1.98)	
Yes	175	71(40.6)	0.61 (0.41,0.89)	
Diabetes				0.582
No	201	77(38.3)	0.70 (0.50,0.97)	
Yes	71	20(28.2)	0.85 (0.45,1.59)	
NIHSS (Median)				0.526
<15	132	35(26.5)	0.63 (0.39,1.02)	
≥15	140	62(44.3)	0.83 (0.57,1.23)	
Aspect (Median)				0.310
<8	111	53(47.7)	0.66 (0.43,1.01)	
≥8	161	44(27.3)	0.81 (0.53,1.23)	
Glucose (Median)				0.058
<7.5	134	48(35.8)	0.58 (0.37,0.89)	
≥7.5	138	49(35.5)	1.04 (0.68,1.60)	
CREA				0.582
<67	131	47(35.9)	0.79 (0.49,1.27)	
≥67	141	50(35.5)	0.72 (0.48,1.08)	
UREA				0.857
<5.06	134	57(42.5)	0.73 (0.47,1.12)	
≥5.06	138	40(29.0)	0.94 (0.61,1.45)	
TICI				0.029
Lv 3	196	62(31.6)	0.64 (0.45,0.91)	
Lv 2b	76	35(46.1)	1.31 (0.71,2.42)	

If not stratified, adjusted for “Hypertension,” “TICI,” “NIHSS,” “Aspect”.

#### 3.4.2 Interaction analysis when UA level is used as a categorical variable

When UA levels were categorized using a clinical threshold of 360 μmol/L as a categorical variable, it was found that patients with low blood glucose levels (< 7.5 mmol/L) and higher UA levels (UA ≥ 360 μmol/L) had a significantly lower risk of moderate-to-severe CED (aOR: 0.24; 95% CI, 0.09–0.69; *p* = 0.033). Similarly, patients with a history of hypertension and those in the high UA level group (UA ≥ 360 μmol/L) exhibited a reduced risk of moderate-to-severe CED (aOR: 0.35; 95% CI, 0.16–0.79; *p* = 0.04). Furthermore, no significant interaction was observed for the remaining variables, including age, gender, history of diabetes mellitus, NIHSS score, ASPECT score, mTICI classification, creatinine, and urea (multivariate-adjusted interaction *p* > 0.05), as outlined in Table 4.

**Table 4:** Interaction analysis between significant variables when uric acid level is used as a categorical variable.

Subgroup	UA<360		UA> = 360			P for interaction
	*N*	Severe CED (%)	N	Severe CED (%)	OR (95%CI)	
Age						0.428
<70	95	39(41.1)	35	6(17.1)	0.37 (0.13,1.02)	
≥70	105	39(37.1)	37	13(35.1)	0.59 (0.24,1.45)	
Gender						0.487
Male	111	44(39.6)	48	13(27.1)	0.61 (0.28,1.37)	
Female	89	34(38.2)	24	6(25.0)	0.38 (0.12,1.24)	
Hypertension						0.040
No	76	20(26.3)	21	6(28.6)	1.27 (0.41,3.94)	
Yes	124	58(46.8)	51	13(25.5)	0.35 (0.16,0.79)	
Diabetes						0.646
No	143	61(42.7)	58	16(27.6)	0.54 (0.26,1.14)	
Yes	57	17(29.8)	14	3(21.4)	0.36 (0.08,1.75)	
NIHSS (Median)						0.711
<15	105	31(29.5)	27	4(14.8)	0.39 (0.12,1.29)	
≥15	95	47(49.5)	45	15(33.3)	0.59 (0.27,1.32)	
Aspect (Median)						0.678
<8	85	44(51.8)	26	9(34.6)	0.51 (0.19,1.39)	
≥8	115	34(29.6)	46	10(21.7)	0.48 (0.19,1.17)	
Glucose (Median)						0.033
<7.5	94	41(43.6)	40	7(17.5)	0.24 (0.09,0.69)	
≥7.5	106	37(34.9)	32	12(37.5)	1.06 (0.43,2.59)	
CREA						0.724
<67	111	43(38.7)	20	4(20.0)	0.38 (0.11,1.36)	
≥67	89	35(39.3)	52	15(28.8)	0.61 (0.27,1.36)	
UREA						0.926
<5.06	113	51(45.1)	21	6(28.6)	0.47 (0.15,1.41)	
≥5.06	87	27(31.0)	51	13(25.5)	0.73 (0.30,1.80)	
TICI						0.264
Lv 3	140	50(35.7)	56	12(21.4)	0.46 (0.21,1.01)	
Lv 2b	60	28(46.7)	16	7(43.8)	0.83 (0.24,2.89)	

If not stratified, adjusted for “Hypertension,” “TICI,” “NIHSS,” “Aspect”.

### 3.5 Correlation between UA and CED at different blood glucose levels

A graph depicting the correlation between UA and CED at varying blood glucose levels demonstrated non-linear inverse trends. Interestingly, the curves appeared more pronounced in patients with low blood glucose levels (< 7.5 mmol/L) compared to those with high blood glucose levels (≥ 7.5 mmol/L). This observation suggests that the negative correlation between UA levels and CED was stronger in patients with low blood glucose levels (< 7.5 mmol/L) (Supplementary Figure S2).

### 3.6 Correlation curves of UA and CED in patients with different histories of hypertension

A graph illustrating the correlation between UA and CED among patients with varying histories of hypertension revealed a non-linear inverse trend. Notably, this curve appears more pronounced in patients with a history of hypertension compared to those without such history. This observation suggests that stronger negative correlation is present between UA levels and CED in patients with a history of hypertension (Supplementary Figure S3).

## 4 Discussion

Numerous studies have highlighted the neuroprotective role of UA in patients with AIS undergoing intravenous thrombolysis (10). A meta-analysis involving 8,131 participants revealed that patients with AIS with elevated UA levels at onset exhibited improved prognosis (11). However, limited research has investigated the association between UA levels and CED in patients with LVO-AIS undergoing MT.

This study elucidated an inverse correlation between UA levels and risk of CED in patients with LVO-AIS undergoing MT (aOR = 0.74; 95% CI, 0.56–0.99; *p* = 0.044). Additionally, the inverse association between UA and moderate-to-severe CED was notably stronger in individuals with lower blood glucose levels at baseline. In essence, higher UA levels post-MT may serve as a protective factor against CED development in patients with LVO-AIS. Moreover, this potential protective effect was particularly prominent in patients with lower blood glucose levels.

Reperfusion therapy plays a crucial role in restoring cerebral blood flow and mitigating tissue damage (3) However, both edema and hemorrhage can result from the gradual increase in BBB permeability and damage. Elevated BBB permeability is associated with hemorrhagic transformation (HT) and CED (12, 13). UA, as an endogenous antioxidant, could scavenge reactive oxygen species, thereby reducing BBB permeability and damage and ultimately lowering the incidence of moderate-to-severe CED. It is important to distinguish this acute neuroprotective role of UA, reflected in its rapid consumption as an antioxidant during ischemia–reperfusion, from the chronic vascular risk associated with sustained hyperuricemia, which operates through distinct long-term pathophysiological mechanisms. Investigating the functional mechanism of UA levels in these patients may aid in identifying those at high risk of CED. Previous studies have demonstrated that higher blood glucose levels are linked to unfavorable functional outcomes, HT, and increased mortality in patients with AIS undergoing intravenous or intra-arterial thrombolysis (14–16). Additionally, a history of diabetes mellitus and elevated blood glucose levels have been associated with poor functional prognosis at 3 months post-mechanical thrombectomy in patients with AIS (17). The mechanisms underlying the association between hyperglycemia and poor prognosis in patients with AIS include altered BBB permeability, increased lactate production in ischemic tissues, impaired cerebrovascular reactivity, and heightened susceptibility to reperfusion injury (18, 19).

Although not statistically significant, a higher NIHSS at presentation of patients who eventually developed moderate to severe CED is described. Moreover, the lower recanalization score, lower ASPECTS score and higher rates of hypertension seen in the moderate-to-severe CED group, all of which reached statistical significance, are more likely contributors to the development of a larger ischemic “core” which would result in higher rates of moderate to moderate-to-severe CED.

Two vital metabolic indicators, blood glucose and UA levels, may influence each other. Considering these findings, glucose availability may increase upon reperfusion of the ischemic territory, leading to heightened formation of free radicals in the ischemic penumbra (20, 21). UA administration effectively scavenges free radicals generated by peroxynitrite, thereby mitigating glucose toxicity and secondary cell death (22, 23). Mounting evidence suggests that damage to the BBB underlies the pathophysiology of hypertension, with acute blood pressure elevation hypothesized to directly impact BBB permeability and increase HT (24, 25). This discovery sheds light on the intricate relationship between UA and hypertension risk, highlighting the importance of maintaining cellular metabolic homeostasis post-cerebral ischemia. Moreover, ischemia-induced inflammatory responses may exacerbate damage to the neurovascular unit, a key pathogenic mechanism in hypertension (26, 27).

In our study, we observed a stronger negative association between UA levels and CED in patients with a history of hypertension (multivariate-adjusted interaction *p* = 0.02), suggesting that the potential protective effect of higher UA levels on CED is more pronounced in these patients. However, research exploring the impact of serum UA levels on hypertension remains limited, leaving unanswered questions concerning its role in hypertension. Further investigation is warranted to elucidate the potential neuroprotective mechanisms of serum UA and its relationship with hypertension.

The primary therapeutic goal of mechanical thrombectomy is to salvage tissue at risk, minimizing the risk of malignant infarctions in patients with low ASPECTS. The observed effect of vessel recanalization on edema progression directly correlates with favorable clinical outcomes, including a reduced incidence of malignant infarctions and improved mRS scores (28).

The findings from our study indicate that patients in the moderate-to-severe CED group exhibited lower mTICI classifications (*p* < 0.05). Moreover, we observed a stronger negative association between UA levels and CED in patients with higher mTICI classifications (grade 3) at baseline (multivariate-adjusted interaction *p* = 0.029). This suggests that the potential protective effect of higher UA levels against CED was more prominent in patients with higher mTICI grades.

This phenomenon could be attributed to the fact that serum UA serves as the most abundant endogenous antioxidant in human plasma. It shields the brain from oxidative damage by scavenging hydroxyl radicals and reducing permeability of the BBB. Patients with lower mTICI classifications typically experience lower rates of recanalization and reperfusion. Consequently, the BBB’s susceptibility to injury induced by reperfusion is also increased.

## 5 Conclusion

There is an inverse correlation between UA levels and risk of CED in patients with LVO-AIS undergoing MT. Elevated levels of UA might shield against CED following mechanical clot removal in patients with LVO-AIS. This correlation between UA levels and risk of CED was more pronounced in individuals with lower blood glucose levels, higher grades of mTICI, and a history of hypertension. In this study, we investigated and analyzed factors influencing CED after mechanical clot removal, focusing on cerebral blood flow. We offer a fresh perspective on the analysis of factors contributing to CED post-clot removal. Such investigations will enhance our understanding of the connection between UA and cerebrovascular disease, laying a solid foundation for future strategies in preventing and treating CED.

This single-center retrospective study has several limitations. First, the relatively small sample size may have increased the risk of statistical error and introduced selection bias, potentially affecting the generalizability of the findings. Second, patients with posterior circulation strokes were excluded from this study; therefore, the findings are applicable only to anterior circulation LVO-AIS and should not be generalized to all stroke subtypes. Third, complete blinding of imaging assessors to serum uric acid levels was not feasible given the retrospective design, which may have introduced subjective bias in edema grading; future prospective studies should incorporate standardized blinded consensus reading by independent neuroradiologists. Fourth, only admission serum UA levels were available; serial measurements at multiple time points during hospitalization were not systematically obtained, precluding a dynamic assessment of the temporal relationship between UA fluctuations and CED progression. Fifth, a uniform UA threshold of 360 μmol/L was applied regardless of sex, which may have introduced misclassification bias; sex-stratified analyses were not feasible given sample size constraints. Sixth, several clinically important covariates were unavailable, including collateral circulation status, bridging thrombolysis, anesthesia strategy, in-hospital blood pressure and glucose trajectories, and hemorrhagic transformation classification, which may have resulted in residual confounding. Finally, reverse causation cannot be excluded, as lower admission UA levels may partly reflect the severity of acute ischemic brain injury rather than a pre-existing risk factor.

In the future, clinical trials investigating the therapeutic effects of UA in AIS patients should consider stratifying specific patient subgroups, such as those with lower randomized blood glucose levels (<7.5 mmol/L), a history of hypertension, or higher mTICI classifications. This approach would enable a more targeted evaluation of UA efficacy across diverse patient populations and inform the design of future clinical trials. Such a design will help to more accurately assess the efficacy of UA in different patient groups and provide a valuable reference for future clinical trial designs. Furthermore, future studies should incorporate serial measurements of serum uric acid at multiple time points during hospitalization, including at admission, 24 h post-thrombectomy, and on day 5 or 7, to better characterize the temporal dynamics of UA and its evolving relationship with CED following mechanical thrombectomy. Through these efforts, we hope to bring new breakthroughs in the treatment of AIS and provide new ideas and methods for the prevention and treatment of CED.

## Data Availability

The raw data supporting the conclusions of this article will be made available by the authors, without undue reservation.

## Supplementary material

SUPPLEMENTARY FIGURE S1 Uric acid and cerebral edema correlation curves. Curves were derived from a Generalized Additive Model (GAM) with restricted cubic splines. The y-axis represents the log odds ratio (log-OR) for moderate-to-severe CED; values below 0 indicate a lower risk of moderate-to-severe CED with increasing UA levels. Shaded areas represent 95% confidence intervals.

SUPPLEMENTARY FIGURE S2 Correlation between uric acid and cerebral edema at different blood glucose levels.

SUPPLEMENTARY FIGURE S3. Correlation curves of uric acid and cerebral edema in patients with different histories Of hypertension.
