# Supplementary figures and images for: Association of uric acid levels with the risk of severe CED in LVO-AIS patients after mechanical thrombectomy
Source: Front Neurol. 2026 May 4;17:1773323. doi: 10.3389/fneur.2026.1773323 (PMC13181920; doi:10.3389/fneur.2026.1773323)

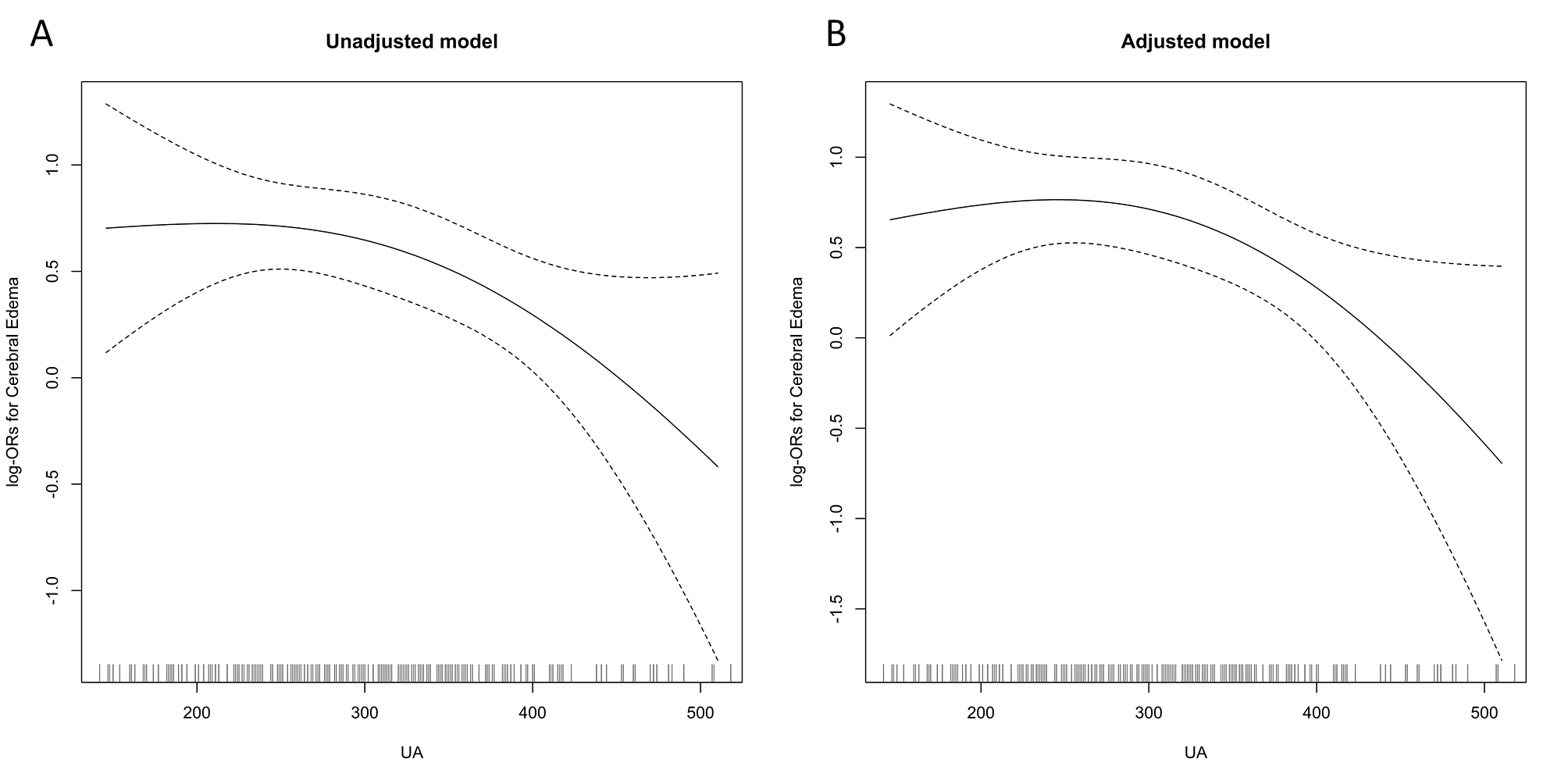

Supplement: SUPPLEMENTARY FIGURE S1 — Uric acid and cerebral edema correlation curves. Curves were derived from a Generalized Additive Model (GAM) with restricted cubic splines. The y-axis represents the log odds ratio (log-OR) for moderate-to-severe CED; values below 0 indicate a lower risk of moderate-to-severe CED with increasing UA levels. Shaded areas represent 95% confidence intervals. [file Image_1.tif]

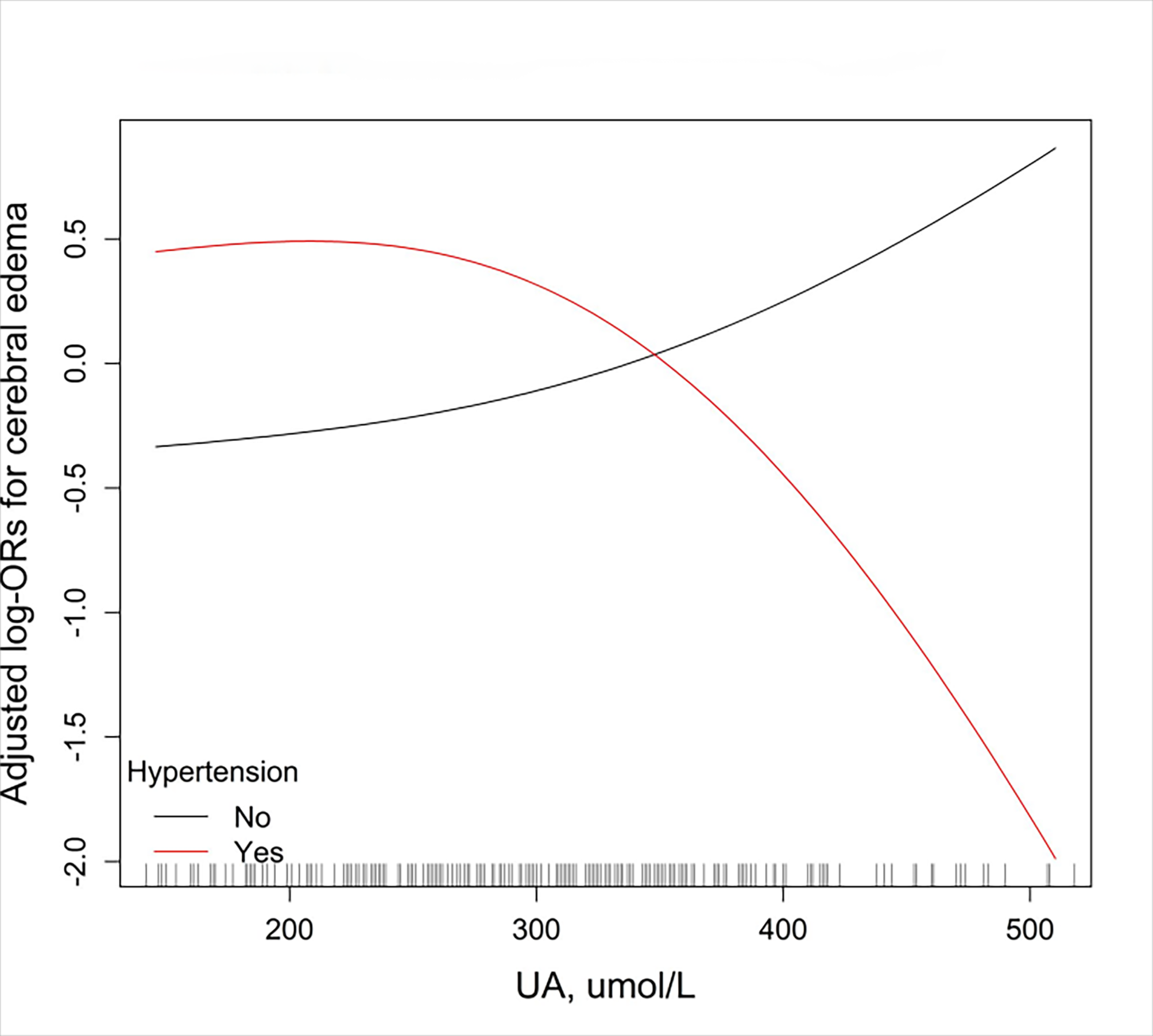

Supplement: SUPPLEMENTARY FIGURE S2 — Correlation between uric acid and cerebral edema at different blood glucose levels. [file Image_2.tif]

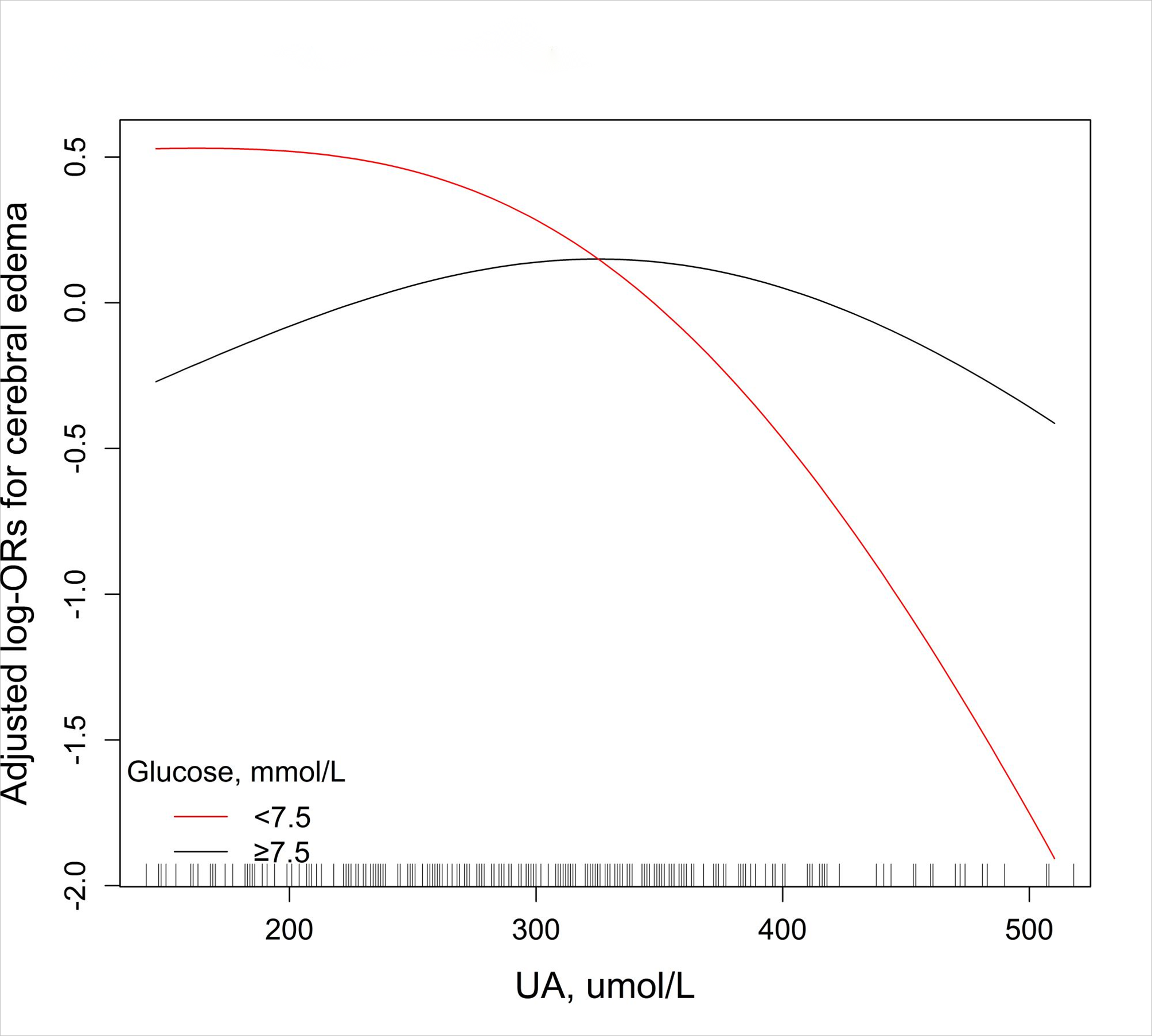

Supplement: SUPPLEMENTARY FIGURE S3. — Correlation curves of uric acid and cerebral edema in patients with different histories Of hypertension. [file Image_3.tif]
